# 5-Aminolevulinic acid-induced severe hypotension during transurethral resection of a bladder tumor: a case report

**DOI:** 10.1186/s40981-019-0279-1

**Published:** 2019-09-04

**Authors:** Tomoaki Yatabe, Shigematsu-Locatelli Marie, Hideo Fukuhara, Takeshi Karashima, Keiji Inoue, Masataka Yokoyama

**Affiliations:** 1 Department of Anesthesiology and Intensive Care Medicine, Kochi Medical School, Kohasu Oko-cho Nankoku, Kochi, 783-8505 Japan; 2 Department of Urology, Kochi Medical School, Kochi, Japan

**Keywords:** 5-Aminolevulinic acid, Bladder tumor, Hypotension

## Abstract

**Background:**

Although 5-aminolevulinic acid (5-ALA) is used for the photodynamic diagnosis of bladder tumors, hypotension is the most commonly observed adverse effect. We present a case of 5-ALA-induced severe hypotension during transurethral resection of a bladder tumor.

**Case presentation:**

A 68-year-old man underwent transurethral resection of a bladder tumor using 5-ALA under general anesthesia. Three hours before anesthesia induction, ALA 20 mg/kg was administered orally. After anesthesia induction, his blood pressure decreased to 47/32 mmHg. Although we used phenylephrine and ephedrine, hypotension persisted at 50/33 mmHg. Bolus administration of noradrenaline slightly increased his blood pressure to 65/39 mmHg. Following this, bolus administration of adrenaline elevated his blood pressure. We decided to perform surgery under continuous administration of adrenaline.

**Conclusions:**

Our case report suggests that anesthesiologists should consider 5-ALA-induced hypotension as a differential diagnosis for hypotension occurring after anesthesia induction. Moreover, ephedrine and phenylephrine might be less effective in treating this condition.

## Background

5-Aminolevulinic acid (5-ALA) is a natural amino acid and a precursor of protoporphyrin IX (Pp IX), the final intermediate in the heme biosynthetic pathway [1]. Exogenous administration of 5-ALA leads to the accumulation of Pp IX in cancer cells, as cancerous cells are deficient in an enzyme that converts Pp IX to heme [2]. Since Pp IX is a photosensitizer and emits a red fluorescence upon excitation with visible blue light, 5-ALA is used for the photodynamic diagnosis of malignant cells, including bladder tumors [3–5]. A previous systematic review reported that hypotension was the most common adverse effect of 5-ALA use [6]. We present here the case of 5-ALA-induced severe hypotension during transurethral resection of a bladder tumor.

## Case presentation

A 68-year-old man (165 cm, 74 kg) was scheduled for transurethral resection of a bladder tumor using 5-ALA. He had been receiving amlodipine 5 mg and azilsartan 20 mg every morning orally for hypertension. His blood pressure was maintained around 130/70 mmHg. Since he had been diagnosed with pyogenic spondylodiscitis in the fourth/fifth lumber disk region in his forties, general anesthesia was preferred. During the pre-operative examination, chest X-ray, electrocardiogram, and laboratory test findings were normal.

The patient took only amlodipine besylate on the morning of the surgery. Three hours before anesthesia induction, 5-ALA 20 mg/kg (Alaglio, Chugai Pharmaceutical, Tokyo, Japan) was administrated orally. Just before anesthesia induction, the patient’s blood pressure was 98/61 mmHg and his heart rate 82 beats/min. Anesthesia was induced with propofol 100 mg, rocuronium 50 mg, fentanyl 100 μg, and remifentanil 0.4 μg/kg/min. After 5 min, just before tracheal intubation, his blood pressure decreased to 47/32 mmHg. There were no observed changes in his electrocardiogram, such as ST-T changes or arrhythmia. Although we used phenylephrine at a total dose of 0.2 mg and ephedrine at a total dose of 10 mg over 10 min, hypotension persisted at 50/33 mmHg. Wheezing and erythema were not observed. Administration of 100 mL hydroxyethyl starch bolus for a few minutes was unsuccessful in increasing blood pressure. Transthoracic cardiac ultrasonography revealed normal systolic function, no asynergy, and normal size of the inferior vena cava. Bolus administration of noradrenaline at a total dose of 30 μg over 6 min slightly increased blood pressure to 65/39 mmHg. Arterial blood gas analysis revealed the following: pH, 7.350; HCO_3_^−^ concentration, 23.2 mmol/L; base excess, 2.3 mmol/L; and lactate concentration, 1.7 mmol/L. Following this, bolus administration of adrenaline at a total dose of 30 μg over 9 min elevated the blood pressure from 53/26 to 127/49 mmHg. We decided to perform surgery under continuous administration of adrenaline. Anesthesia was maintained with sevoflurane and remifentanil. The patient’s blood pressure and heart rate ranged from 72/31 to 94/40 mmHg and from 70 to 85 beats/min, respectively, under the administration of adrenaline at 0.01–0.07 μg/kg/min. On completion of the surgery, he was transferred to the intensive care unit under intubation. Twelve minutes after admission to the intensive care unit, he was extubated as his blood pressure had stabilized at 142/56 mmHg under the administration of adrenaline at 0.03 μg/kg/min. Ten minutes after extubation, adrenaline infusion was discontinued. Although his lactate concentration was 5.3 mmol/L at this time, it decreased to 2.7 mmol/L after 3 h. His blood pressure and heart rate were stable during his intensive care unit stay. The patient was then transferred to the general ward on day 1 postoperatively. He was discharged without any complications 6 days postoperatively. Three months later, dermatologists performed a skin-prick test on him for propofol, rocuronium, and 5-ALA, the results of which were negative. Five months later, he received TUR-Bt under general anesthesia without 5-ALA for recurrence of tumor. Although we used propofol and rocuronium, severe hypotension did not occur.

## Discussion

A previous systematic review reported that hypotension was the most common adverse effect of 5-ALA, since hypotension comprised 60% of these events [6]. A small observational study, which enrolled 20 patients who underwent radical prostatectomy reported that the noradrenalin dose to maintain mean blood pressure within 70–90 mmHg in the 5-ALA group was significantly higher than that in the control group (0.08 ± 0.04 μg/kg/min vs. 0.03 ± 0.02 μg/kg/min; *p* < 0.01) [7]. Another observational study, which enrolled 38 patients who underwent photodynamic diagnostic ureterorenoscopy using 5-ALA, reported that 20 patients developed hypotension after ingestion of 5-ALA [8]. This study also reported that three patients developed symptomatic hypotension preoperatively. Fluid resuscitation with 250 mL colloid and 500 mL saline was successful in increasing blood pressure [8]. Fluid resuscitation might be useful, as the potential mechanism of 5-ALA-induced hypotension might be attributed to the vasodilatory effect induced by Pp IX [7]. A previous retrospective study of 30 patients who underwent photodynamic diagnostic ureterorenoscopy with 5-ALA reported that 13% of patients developed hypotension, and all patients had taken antihypertensive drugs on the day of surgery [9]. The authors suggested that antihypertensive drugs should be discontinued on the day of surgery. Another study evaluated 5-ALA-induced adverse effects in 90 patients and revealed that a history of hypotension and antihypertensive therapy were independent risk factors for 5-ALA-induced hypotension [10]. Our patient had hypertension and was administered two classes of antihypertensive drugs, namely, calcium-channel blockers and angiotensin II receptor antagonists. Furthermore, our patient took a calcium-channel blocker on the morning of the surgery. Although his normal blood pressure was 130/70 mmHg, it decreased to 98/61 mmHg after ingestion of 5-ALA. This suggests that our patient developed 5-ALA-induced hypotension, based on the findings of previous studies.

A recent review reported that it is important to consider anaphylaxis as a differential diagnosis when perioperative hypotension or bronchospasm do not respond to conventional therapy during general anesthesia [11]. We also suspected anaphylaxis caused by propofol or rocuronium, as hypotension did not respond to conventional drugs such as ephedrine and phenylephrine. Hypotension, bronchospasm, angioedema, and dermatological symptoms are common clinical signs of perioperative allergic reactions [11]. In our case, hypotension was the only symptom. Moreover, allergy skin tests for propofol, rocuronium, and 5-ALA were negative. Severe hypotension did not occur in our patient’s subsequent TUR-Bt without 5-ALA. Therefore, we eliminated perioperative allergic reactions as a potential cause of hypotension.

We believe that our case raises two important inferences. First, some patients with 5-ALA-induced hypotension might not respond to conventional therapy, akin to anaphylaxis. As mentioned above, fluid resuscitation and noradrenaline were shown to be effective in previous studies. Bolus administration of 100 mL hydroxyethyl starch and noradrenaline could not recover our patient’s blood pressure, despite the lack of systolic dysfunction and asynergy. Adrenaline should probably be considered in such situations. Second, the use of antihypertensive agents on the morning of the surgery should be reconsidered. Previous studies and our case suggest that a history of hypertension and the use of antihypertensive agents are risk factors of 5-ALA-induced hypotension. From the perspective of balancing the risks and benefits, further detailed studies are required to identify the risk factors of 5-ALA-induced hypotension.

In conclusion, our case report suggests that anesthesiologists should consider 5-ALA-induced hypotension as a differential diagnosis for hypotension occurring after anesthesia induction. Moreover, ephedrine and phenylephrine might be less effective in treating this condition.

## Data Availability

Not applicable.

## Abbreviations

5-ALA: 5-Aminolevulinic acid
Pp IX: Protoporphyrin IX
TUR-Bt: Transurethral resection of a bladder tumor
